# Evaluation of the effectiveness of music therapy in improving the quality of life of palliative care patients: a randomised controlled pilot and feasibility study

**DOI:** 10.1186/s40814-016-0111-x

**Published:** 2016-11-29

**Authors:** Tracey McConnell, Lisa Graham-Wisener, Joan Regan, Miriam McKeown, Jenny Kirkwood, Naomi Hughes, Mike Clarke, Janet Leitch, Kerry McGrillen, Sam Porter

**Affiliations:** 1 School of Nursing and Midwifery, Queen’s University Belfast, Belfast, Northern Ireland; 2 Marie Curie Hospice Belfast, Belfast, Northern Ireland; 3 Every Day Harmony Music Therapy, Belfast, Northern Ireland; 4 School of Medicine, Dentistry and Biomedical Sciences, Queen’s University Belfast, Belfast, Northern Ireland

**Keywords:** Music therapy, Palliative care, End-of-life care, Hospice, Pilot study, Feasibility study

## Abstract

**Background:**

Music therapy is frequently used as a palliative therapy. In consonance with the goals of palliative care, the primary aim of music therapy is to improve people’s quality of life by addressing their psychological needs and facilitating communication. To date, primarily because of a paucity of robust research, the evidence for music therapy’s effectiveness on patient reported outcomes is positive but weak. This pilot and feasibility study will test procedures, outcomes and validated tools; estimate recruitment and attrition rates; and calculate the sample size required for a phase III randomised trial to evaluate the effectiveness of music therapy in improving the quality of life of palliative care patients.

**Methods:**

A pilot randomised controlled trial supplemented with qualitative methods. The quantitative data collection will involve recruitment of >52 patients from an inpatient Marie Curie hospice setting over a 12-month period. Eligibility criteria include all patients with an Eastern Cooperative Oncology Group (ECOG) performance status of 03− indicating they are medically fit to engage with music therapy and an Abbreviated Mental Test (AMT) score of ≥7 indicating they are capable of providing meaningful informed consent and accurate responses to outcome measures. Baseline data collection will include the McGill Quality of Life Questionnaire (MQOL); medical and socio-demographic data will be undertaken before randomisation to an intervention or control group. Participants in the intervention arm will be offered two 30–45 min sessions of music therapy per week for three consecutive weeks, in addition to care as usual. Participants in the control arm will receive care as usual. Follow-up measures will be administered in 1, 3 and 5 weeks. Qualitative data collection will involve focus group and individual interviews with HCPs and carers.

**Discussion:**

This study will ensure a firm methodological grounding for the development of a robust phase III randomised trial of music therapy for improving quality of life in palliative care patients. By undertaking the pilot and feasibility trial under normal clinical conditions in a hospice setting, the trial will result in reliable procedures to overcome some of the difficulties in designing music therapy RCTs for palliative care settings.

**Trial registration:**

Clinicaltrials.gov Identifier: NCT02791048

**Electronic supplementary material:**

The online version of this article (doi:10.1186/s40814-016-0111-x) contains supplementary material, which is available to authorized users.

## Background

Music therapy has increasingly been implemented in a range of healthcare settings, including oncology, dementia and intensive care [1–3]. Music therapy is among the two most common forms of complementary therapy provided in hospices [4], with inclusion of music therapy in palliative care settings having a long tradition stemming back to the 1970s [5]. With the World Health Organisation definition of palliative care as ‘the active total care of patients’ where the ‘control of…psychological, social and spiritual problems is paramount’ [6], widespread provision of music therapy is consonant with the holistic approach which distinguishes palliative care from other areas of healthcare.

In terms of palliative care provision, figures for 2006 show there were 193 specialist inpatient units, 295 home care services, 314 hospital-based services, 234 day care services and 314 bereavement support services across England, Wales and Northern Ireland [7]. While there are no figures available for the existing provision of music therapy in palliative care services as a whole, a survey by Daykin et al. [8] showed that music therapy is still an emerging therapy within cancer care across the UK compared to more established interventions such as art therapy. This survey showed that the majority of music therapy provision was funded externally by the NHS, or in a small number of cases from internal funding. Music therapy was also more likely to be used in settings were complementary and alternative therapies were already in place, suggesting a trend towards the use of music therapy as an additional supportive service rather than replacing existing services. There are currently no guidelines in place for the use of music therapy in palliative care. This highlights the need for a stronger evidence base that takes due cognisance of benefits and risks to help inform future music therapy provision [9].

Music therapy entails the use of music to achieve individual goals in the context of a therapeutic relationship with a professional music therapist [10]. In the UK, music therapists are trained to master’s level and are registered with Health and Care Professions Council as allied health professionals. Music therapy aims to improve the quality of life of palliative care patients by relieving physical and psychological symptoms, facilitating communication and alleviating spiritual or existential concerns [11].

Music therapy approaches range from ‘receptive’ to ‘recreative’ depending on the patient’s level of physical limitations. Receptive approaches are more passive in terms of listening to music played by the therapist, while recreative approaches can involve the patient singing or playing their favourite music alongside the therapist [12]. One of the most common approaches for palliative care patients is legacy work, whereby the therapist helps patients produce a compilation of songs that have meaning for them to leave for family and friends [13]. Irrespective of the approach, music therapy aims to support and improve physical, psychological and existential well-being [14]. With goals mirroring those of palliative care, music therapy research within this population is both necessary and of great importance to supportive palliative care initiatives.

Most palliative care referrals for music therapy are in relation to pain and quality of life [15]. A recent systematic review of randomised controlled trials reported significant improvements in pain experienced by palliative care patients receiving music therapy [16]. Although the most recent Cochrane review on music therapy for end-of-life care, published in 2010, recommended more research of high quality to support the effectiveness of music therapy for improving quality of life [17], there have been no additional high-quality studies evaluating this outcome within the last 5 years [16]. Several challenges to conducting music therapy randomised controlled trials (RCTs) in palliative care have also been identified, including high levels of attrition, the need for flexibility in time commitment and inclusion of family members if desired and provision of a standardised yet tailored intervention [17]. Process evaluation using qualitative methods is recommended for future trials to aid in identifying factors which contribute to or limit the effectiveness of music therapy interventions [17].

Based on promising findings regarding the benefits of music therapy for improving the quality of life, more research is needed to produce robust evidence of effectiveness in the field of palliative care where music therapy is already in widespread use and in high patient demand [18, 19]. Furthermore, given that research in music therapy and in palliative care are fraught with challenges [20, 21], pilot and feasibility studies are required to establish the most robust study design and processes to facilitate high-quality research in this area [22].

## Methods/design

### Trial aims and objectives

This study aims to establish the feasibility of and to pilot an RCT to evaluate the effectiveness of music therapy in improving the quality of life and inter-familial communication in palliative care patients. The trial will be conducted under normal clinical conditions to test procedures, outcomes and validated tools; estimate recruitment and attrition rates; and calculate the sample size required for a phase III randomised trial. Qualitative data collection with carers and health care practitioners (HCPs) will supplement this further by examining if music therapy leads to an improvement in inter-familial communication and determining which processes help or hinder the implementation of music therapy within a hospice setting. This protocol follows the SPIRIT guideline for writing protocols (see Additional file 1, SPIRIT 2013 Checklist).

### Primary outcome

The main aim of the study is to evaluate the feasibility of administering the McGill Quality of Life Questionnaire (MQOL) [23], reported to have the best clinometric quality rating, content validity, construct validity and internal consistency of reviewed quality of life questionnaires [24]. We will also assess the viability of delivering a 3-week music therapy intervention, along with using recruitment and attrition rates to determine the sample size required for a phase III randomised controlled trial.

### Secondary outcomes

1. The potential effectiveness of music therapy upon quality of life at week one (one to two music therapy sessions), week three (after completion of the music therapy course) and week five (two weeks after completion of music therapy)
2. The effect of music therapy upon inter-familial communication
3. The effect of contextual factors upon the implementation and sustainability of music therapy in a palliative care setting

### Intervention

Participants will be randomly assigned to two groups. Patients assigned to the control group will receive usual care only from the hospice multidisciplinary team. The dose and frequency of usual care will be as deemed appropriate by the hospice practitioner in charge of their treatment. As music therapy is not part of usual care within this hospice, the control group participants will be offered two sessions of music therapy upon completion of their time in the study. The researcher will notify the music therapist each time a participant has finished their third and final study visit. The music therapist can then offer those in the control group their music therapy sessions when a slot becomes available (see the participant flow chart in Additional file 2).

In addition to the usual care described above, patients assigned to the experimental group will receive music therapy in an individual setting, delivered by a trained music therapist. Music therapy will be conducted for up to 45 min twice a week. A total of six sessions will be offered, with the aim of completing at least four sessions. Patient’s needs will be identified by the music therapist during the first session, guided by preliminary discussions with the hospice clinical team and by the patient’s own assessment of what needs are most important to them. The musical equipment used will include accessible instruments provided by the music therapist.

As recommended by our PPI (Patient and Public Involvement) collaborators on this protocol, patients can choose to have their carer present during the intervention as time together is precious. Patient’s written informed consent to have their carer present will be obtained by the hospice clinician during recruitment. Carers will only be observing the music therapy sessions and will not be directly involved.

In palliative care, the music therapeutic approach used is a creative process of musical interaction where the client engages while singing or playing, listening to music or extemporaneously creating a melody, rhythm, song or instrumental piece. In the sessions, the music therapist uses music in various formats to meet the patients’ specific needs. In doing so, they ‘make use of the therapeutic relationship established with the patient to meet clinical goals and employ a systematic therapeutic process that includes assessment, treatment and evaluation’ [17].

In terms of ‘dose’ of music therapy, previous studies in palliative care patients have shown that a short course, consisting of two or more sessions, is effective and appropriate for this population [25, 26]. While the content of the sessions will be influenced by each patient’s needs, interests, preferences and energy level and adapted accordingly in the moment, the music therapist will complete an intervention manual [27] at the end of each session, which will include the details on who chose the music and what strategy was used (i.e. music listening, active music-making, improvising, legacy work), along with strategies to enhance treatment fidelity (how the intervention was monitored for consistency). This will aid transparent reporting, along with guiding the development of a standardised protocol for use across sites in the main trial.

As with the control group, the dose and frequency of usual care will be as deemed appropriate by the hospice professional in charge of their treatment.

### Study setting

Participants will be recruited from the inpatient unit and day hospice at an 18-bed specialist palliative care unit in Northern Ireland.

### Trial design

A UK-based, single-centre, pilot RCT will involve two parallel arms, one receiving individual music therapy in addition to usual care, and the other receiving usual care only. It is not possible to blind patients to treatment allocation, but the researcher administering the study measures will be blinded up until the completion of the first follow-up visit; after which time, the researcher will invite patients and carers to talk freely about their experience of music therapy if they have been in the intervention arm of the trial. In order to maintain a level of blinding, we propose to have an outcome adjudicator conduct a data check of data entry and a statistician conduct data analysis, both blinded to treatment allocation. As only the researcher will be blinded to the intervention, we cannot foresee circumstances which will require unblinding. This study will employ mixed methods to meet all study objectives.

#### Inclusion criteria for patients

Eligibility will be assessed by a hospice clinician during inpatient admission or day hospice attendance using the Eastern Cooperative Oncology Group (ECOG) scale and the Abbreviated Mental Test (AMT). The ECOG [28] scale is most frequently used by physicians for assessing palliative care patients’ level of functioning [29]. The AMT [30] is highly correlated with the Mini-mental State Examination (MMSE), which has good validity and is widely used to screen for cognitive impairment within a palliative care population [31]. Patients will be eligible if they have:

- An ECOG performance status of 0, 1, 2 or 3 (0 indicating the patient is asymptomatic, 1 the patient is symptomatic but fully ambulatory, 2 the patient is symptomatic and confined to bed for less than 50% of the day and 3 the patient is symptomatic and confined to bed for more than 50% of the day) indicating they are able to engage with interactive music therapy
- A score of 7 or more on the AMT, indicating they are capable of providing meaningful informed consent and accurate responses to the study’s primary outcome measurement tool. Patients with communication difficulties will also be eligible if they are able to indicate their responses to the questionnaire

### Patient exclusion criteria

- ECOG performance status of 3 or 4 (3 indicating the patient is symptomatic and confined to bed for more than 50% of the day, 4 indicating the patient is severely symptomatic and completely bedridden)
- A score of 6 or less on the AMT, indicating they may not be capable of providing fully informed consent or accurate responses to the study’s primary outcome measurement tool
- Participants who decide not to consent will be excluded from the trial. Patients will be assured that this decision will have no implications for the care that they receive

### Allocation and randomisation

Eligible patients who have provided consent will be randomly allocated to the intervention or control arm using a 1:1 allocation ratio. Allocations will be randomly assigned using blocked randomisation with randomly permuted block sizes. Opaque randomisation envelopes will be stored at the hospice in a locked filing cabinet, accessible only to an administrator. To ensure the researcher remains blind, the administrator will forward the treatment allocation (control or experimental group) to the music therapist(s) delivering the intervention.

### Ethics

Patients will be asked for their written informed consent to audio record sessions during their first study visit. In line with best practise (British Association of Music Therapy (BAMT) [32], audio records are used to enhance the therapeutic process by providing the music therapist with an assessment tool to gage how the session went and plan for further sessions. Patients will be informed in their information sheets before the consent is taken that they have the right not to consent to audio recordings of their sessions without having to give a reason and without this affecting their care in any way.

Due to the highly emotive nature of working with a palliative care population, the music therapist delivering the intervention will receive clinical debriefing from their clinical supervisor. The music therapist will also have the opportunity for debriefs with the clinical principal investigator on this study. If the clinical principal investigator feels the music therapist needs additional support, they will signpost them to appropriate services.

The intervention will be discontinued if patients no longer wish to or do not feel able to continue music therapy sessions. The intervention will also be discontinued if hospice practitioners deem that the patient’s changing condition means that music therapy is no longer feasible. In order to promote retention and follow-up, the intervention will be modified to accommodate inpatients who are discharged before their third week of music therapy. In this instance, they will be offered their final week’s sessions as an outpatient if they are medically fit/mobile.

### Sample size

The aim is to recruit 52 patients across both treatment allocations (33% more than the minimum recommended for good practise [33] including a 30% attrition allowance as recommended for a palliative care population [34]). The calculation of the sample size was informed both by good practise for feasibility trials and by our expectations on our ability to recruit eligible patients with the time and funding available for this feasibility trial. As noted, we are hoping to exceed the recommended number for a feasibility trial in order to maximise the information we can gather with the resources available.

### Progression criteria

We will use criteria based on those that we have used in planning the progression from other feasibility studies. These consider the proportion of the target sample size that is achieved during the feasibility study:

- 75–100% recruitment: progress to application for main trial with little or no change to relevant aspects of the protocol
- 50–74% recruitment: progress to application for main trial following a review of details on patients who were not deemed eligible or decline to join the trial, an assessment of any barriers to recruitment and possible changes to relevant aspects of the protocol
- 25–49% recruitment: progress to application for main trial if a ‘rescue plan’ can be developed by the trial team and other potential co-applicants. This plan might include the need to identify additional sites or changes to the relevant aspects of the protocol

Less than 25% recruitment: the trial will probably not progress

### Analytical methods

#### Primary outcomes

The feasibility of using the MQOL questionnaire will be evaluated by the researcher at each data collection point through feedback from patients on the questionnaire’s appropriateness and level of burden. The number of completed questionnaires will also be monitored along with reasons for non-completion. The viability of delivering a 3-week music therapy intervention will be assessed by the number of patients completing the intervention along with reasons for attrition. Recruitment and attrition figures will be used to conduct a power calculation. This will be conducted by the Northern Ireland Clinical Trials Unit to determine the sample size required for a phase III randomised controlled trial.

#### Secondary outcomes

To assess the potential effectiveness of music therapy, data will be analysed using the Statistical Package for Social Sciences, v20. The change in the outcome variable from baseline to 1 week, 3 weeks and 5 weeks, will be compared between the experimental and control group using analysis of covariance [35]. To detect differences within groups, repeated measures ANOVAs will be utilised. As this pilot study does not include a formal power calculation, confidence intervals will be clearly stated with any estimates, and findings should be interpreted with caution. The principle of intention to treat analysis will be performed blind to treatment allocation. In line with the intention-to-treat principle, patients who attend fewer sessions will not be excluded from data analysis. Qualitative methods, in particular interviews and focus groups, will be used to assess inter-familial communication and contextual factors.

### Data management

Data will be collected on a study-specific case report form (CRF) starting at the time of recruitment. This form will collect socio-demographic data at baseline, along with details of current treatments and medications which will be collected at all four study visits—randomisation (baseline), week one (after one to two music therapy sessions), week three (after final music therapy session) and at week five (end of trial). Data will be entered anonymously onto an encrypted file server which will be stored in a secure office. Recommendations for managing attrition and access data when conducting palliative care research will be followed [34]. Different types of attrition will be reported to include attrition due to death (ADD), attrition due to illness (ADI) and attrition at random (AAR). Consideration will also be given to how timing of data collection affects attrition rates. Qualitative data will be managed using the Nvivo V.11 software.

### Data monitoring and interim analyses

This study does not require a data monitoring committee as it will recruit and follow up over a short period of time and the likely risks of harm are minimal. No interim analysis will be planned as the sample size is small, and the study is of a short duration.

### Harm

As music therapy is non-invasive and non-intrusive; no serious adverse events attributed to music therapy have been reported or are expected.

### Qualitative data collection

(i) Carer interviews

Design

Carer interviews aim to assess whether music therapy improves inter-familial communication within a palliative care population. In the absence of a validated tool to measure this outcome, an interview topic guide will be developed by the research team. Interviews will be digitally recorded and transcribed verbatim, at which point recordings will be deleted. Confidentiality will be ensured by removing all identifying information from the transcripts.

Carers who are newly bereaved during the trial will be excluded from the study. Sample size will be dictated by data saturation though it is estimated that 26 carers will be recruited. Carers who chose to sit in on the music therapy sessions will be given an invitation letter and information sheet by the music therapist after the patient’s second music therapy session (end of week one). Carers will also have the opportunity to take part in another interview after the patient’s final music therapy session (week three) if they wish to. For those carers who chose not to sit in on music therapy sessions, an invitation letter and information sheet will be left with their loved one to give to them on their next visit.

Carers who return a Notification of Interest Form to the music therapist or member of hospice staff will be contacted by the researcher and given the opportunity to ask questions about the study, at which point they can decide to opt out or provide written informed consent.

Interviews will take place at the participant’s home if they choose, or in a private room at the hospice. It is anticipated that the interview will last for 40–60 min. The same ethical and data analysis procedures will be conducted as outlined for focus group data. If carers have a concern or become upset about any aspect of the study, they can speak to the researcher or one from the study team. If appropriate, they will ensure that the carer will be given the opportunity to talk any issues through with the hospice care team.

(ii) Process evaluation

Design

Focus groups with HCPs aim to examine contextual factors and subgroup response to the intervention and acceptability. A semi-structured interview guide will be utilised with the focus groups moderated by an experienced qualitative researcher. The focus group topic guide will be based on realistic evaluation principles to advance theoretical understanding of what components of music therapy work best, for whom and in what circumstances [36]. Focus groups will be digitally recorded and transcribed verbatim, at which point recordings will be deleted. Anonymity will be ensured by removing all identifying information from the transcripts.

Setting and recruitment

Using a purposeful sampling approach, HCPs with a direct patient role will be recruited from the hospice, approximately 2 months after trial initiation. The sample size will be dictated by data saturation, with an estimated 16 HCPs to be recruited. Potential participants will be sent invitation letters and information sheets via email by an administrator at the hospice. Written informed consent will be secured prior to commencing focus groups. An experienced qualitative researcher will conduct the focus groups to ensure rigour.

Focus groups will take place in a private room at the hospice and will last approximately 1 h. Data will be analysed by two researchers using a thematic content analysis based on Newell and Burnard’s framework [37] and informed by a realist approach as outlined by Porter [38] to enhance both rigour and the ‘confidence criterion’ (the level of confidence practitioners have that the findings presented accurately portray and explain the issues being addressed and will inform their practise) (p. 83).

### Auditing

The research team will meet monthly to discuss study progress. Monthly auditing of recruitment will be generated and distributed to the Trial Steering Committee for discussion at its meetings.

### Protocol amendments

Any protocol amendments will be discussed firstly with the university’s Research Governance to assess if they constitute a minor or major amendment. Approval will then be sought from the ORECNI before any amendments are implemented.

### Confidentiality

All members of the study team will be trained and adherent to participant confidentiality in line with good clinical practise. All procedures relating to the research will be conducted in secure clinical settings within the hospice, with home options for carer interviews. No identifiable information about participants will be used in any report or publication, and participants will be informed that any digital recordings will be destroyed upon completion of the research. The personal data from interviews will be anonymised using a unique identifier code number.

### Access to data

Only those in the research team will have access to the data.

### Post-trial care

All patients will continue to receive usual care at the hospice during and after the study. Patients, carers, and practitioners will be reminded that if they have a concern or become upset about any aspect of this study, they can speak to the researcher or one of the study team.

### Dissemination

Marie Curie Hospice, Every Day Harmony Music Therapy and Queen’s University Belfast websites will disseminate information on involvement in trial. The trial results will be communicated via conference presentations, peer-reviewed publications and the aforementioned organisations’ websites in order to reach participants, healthcare professionals and the public.

## Discussion

This study is designed to establish the feasibility of and to pilot a randomised controlled trial to evaluate the effectiveness of music therapy in improving the quality of life of palliative care patients. The findings will be valuable for estimating recruitment and attrition rates to inform the sample size required for a phase III multi-site randomised trial. Findings from the feasibility components will also help ascertain if the instruments for measuring the outcomes constitute an acceptable burden for participants and whether attrition affects the viability of an intervention of 3 weeks duration. If music therapy is found to be effective for improving the quality of life of palliative care patients in a subsequent phase III RCT, this finding will provide support for NHS and third sector specialist palliative care commissioners and service providers to make an evidence-based decision on whether to incorporate music therapy in palliative care services, with patients as beneficiaries.

For this study, anticipated challenges include generating and maintaining enthusiasm among practitioners referring potential patients to the study. Strategies to overcome these challenges will include research training sessions for practitioners at the hospice, research team attendance at multidisciplinary team meetings and monthly newsletters updating on study progress [20]. An important issue to consider in research in general and for palliative care research in particular is Personal and Public Involvement (PPI). Marie Curie’s Expert Voices Group was instrumental in shaping this protocol and will continue to be involved in trial governance throughout the project.

## Additional files

Additional file 1: SPIRIT 2013 Checklist: Recommended items to address in a clinical trial protocol and related documents*. (DOC 123 kb)
Additional file 2: Participant Flowchart. (DOCX 35 kb)

## Abbreviations

AAR: Attrition at random
ADD: Attrition due to death
ADI: Attrition due to illness
AMT: Abbreviated Mental Test
CRF: Case Report Form
ECOG: Eastern Cooperative Oncology Group
HCP: health care practitioner
MQOL: Mcgill Quality of Life Questionnaire
ORECNI: Office for Research Ethics Committees Northern Ireland
PPI: Personal and Public Involvement
RCT: Randomised controlled trial
SAE: Serious adverse event
TSC: Trial Steering Committee
