# Supplementary figures and images for: Evaluation of the effectiveness of music therapy in improving the quality of life of palliative care patients: a randomised controlled pilot and feasibility study
Source: Pilot Feasibility Stud. 2016 Nov 29;2:70. doi: 10.1186/s40814-016-0111-x (PMC5154028; doi:10.1186/s40814-016-0111-x)

Participant Flowchart


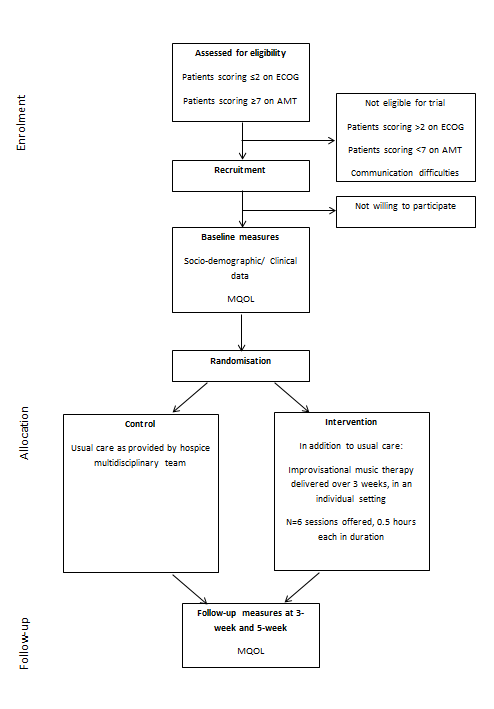

Supplement: Additional file 2: — Participant Flowchart. (DOCX 35 kb) [file 40814_2016_111_MOESM2_ESM.docx]
